# American Academy of Dental Science, *January 4, 1905*

**Published:** 1905-12

**Authors:** 


### AMERICAN ACADEMY OF DENTAL SCIENCE,

*January 4, 1905.*

### ADJUSTING CLASPS TO PARTIAL DENTURES.

*Dr. Werner.*—Mr. President and Fellows of the Academy:—I don't know that I know the best way to make these adjustments. But occasionally we make plates. At least I do. And occasionally we make difficult plates. At least I have to. About a month ago this rather difficult case, to get an accurate impression of and to adjust a partial plate nicely, came into my hands, and how did I do it? That is what I am going to tell.

First, I was careful in getting an accurate impression. Then I spent about an hour in trying that plate in, bending it and burnishing it, rebending it and reburnishing it, until I thought I had it as good as I could make it. Second, I took impressions of the three teeth to be clasped and had the clasps made.

In a lower partial case where there are two teeth on the left and one tooth on the right to be clasped and where there is a space between the central incisors, the first important thing is to make a plate so low on the inside as not to come up to the teeth and show through the spaces, and that means a narrow, thick, strong plate, a plate of two thicknesses of No. 28 or 29 gage. Next was to adjust the clasp to the most difficult of the teeth to be clasped, which in this case was the left inferior third molar, which tooth was tipped at an angle of about forty-five degrees.

We all believe in having clasps adjusted so as to do the least harm. Now, the harm by clasps is done by molecular attraction and retention. If your clasp fits very well all around, it has the greatest molecular retention and attraction. A clasp need not necessarily fit like a glove. Adjust that clasp to the most difficult of the teeth to be clasped and hold it there with an excavator till you have it fixed. With a little sticky wax, at once take the impression of the tooth and clasp with modelling compound. The warm modelling compound will dissolve the wax that the clasp was stuck by to the teeth, and it will come off in exactly the position wanted. That clasp is then soldered to the plate. Then I proceed with two other clasps the same way—the one on the outside and the other on the inside.

DISCUSSION.

*Dr. Horatio C. Meriam.*—A good impression for partial cases, when an impression of one side of the mouth only is needed, can be taken by making a small cup of air-chamber metal and cutting holes to let the remaining teeth pass through. A rough impression is modelling compound may be taken first, then removed, cooled and trimmed. Then add a layer of soft modelling compound and replace in the mouth. A very sharp impression of the space between molar and cuspid may be made in this way.

Dr. Bonwill some years ago described a clasp that touched the tooth only on three points, and a few years ago Dr. Fred. Bogue gave in New York a description of some improved wire clasps. I am reminded to-night of the late Dr. Chandler, who insisted that we should not say capillary attraction, for that came from the word *capillus,* a hair. "It is adhesive attraction!" Professor Chandler had been a teacher in the Latin school and often gave us a touch of the schoolmaster.

*Dr. Werner.*—I know the way in which Dr. Stoddard and Dr. Dickinson do it, but it is, generally speaking, that way in which you do it well that is the best for you or for me. I was not speaking to-night so much of impressions.

*Dr. Stoddard.*—I have followed Dr. Werner's method of adjusting clasps to plates for a good many years, except using soft plaster instead of modelling compound to get the relation. Another method which I use in partial gold cases is, get an accurate impression with plaster of paris and when the model is being cast pour soft plaster into the teeth and put a flat pin into them, allow it to harden, and then pour the balance of the model. When it is hard the teeth may be readily removed from the model and the plate struck up and the teeth put back in position and the clasps fitted to them, so the adjustment is absolutely accurate.

